# *Heterostemma cucphuongense* (Apocynaceae, Asclepiadoideae), a new species from Vietnam

**DOI:** 10.3897/phytokeys.148.50029

**Published:** 2020-05-26

**Authors:** The Bach Tran, Le Ngoc Han, Do Van Hai, Bui Hong Quang, Nguyen Thi Thanh Huong, Bui Thu Ha, Tran Van Hai, Michele Rodda

**Affiliations:** 1 Department of Botany, Institute of Ecology and Biological Resources, Vietnam Academy of Science and Technology, 18 Hoang Quoc Viet, Cau Giay, Hanoi, Vietnam Graduate University of Science and Technology Hanoi Vietnam; 2 Graduate University of Science and Technology, Vietnam Academy of Science and Technology, 18 Hoang Quoc Viet, Cau Giay, Hanoi, Vietnam Institute of Ecology and Biological Resources, Vietnam Academy of Science and Technology Hanoi Vietnam; 3 Hanoi National University of Education, 136, Xuan Thuy Street-Cau Giay District, Hanoi, Vietnam Hanoi National University of Education Hanoi Vietnam; 4 Singapore Botanic Gardens, National Parks Board, 1 Cluny Road, Singapore, 259569, Singapore Singapore Botanic Gardens, National Parks Board Singapore Singapore

**Keywords:** Ceropegieae, Cuc Phuong National Park

## Abstract

*Heterostemma cucphuongense* (Apocynaceae, Asclepiadoideae), a new species from Vietnam is described, illustrated and compared with the similar species *Heterostemma succosum* Kerr. *Heterostemma cucphuongense* differs from *H. succosum* by the morphology of the rachis of the inflorescence, the margins of the corolla lobes and the colour of the adaxial surface of the corolla.

## Introduction

The genus *Heterostemma* Wight and Arn. comprises approximately 30 to 40 species and is widely distributed from India and China to Australia and the Western Pacific Islands (Li et al. 1995; Swarupanandan et al. 1989; Forster 1992). The number of species has increased in recent years and three new species were published in 2019 alone. These are: *Heterostemma barikiana* P.Agnihotri et al. from India, Myanmar and Thailand, (Agnihotri et al. 2019), *H. ficoides* A.Kidyoo (Kidyoo 2019) from Thailand and *Heterostemma trilobatum* A.Kidyoo & Thaithong also from Thailand (Kidyoo and Thaithong 2019). However, this third species is indistinguishable from *H. barikiana*. Furthermore, the specimen *C. Maknoi & P. Srisanga 2258* (QBG) was cited under both *H. barikiana* and *H. trilobatum* and, therefore, *H. trilobatum* should be considered as a synonym of *H. barikiana*.

For Vietnam, extensive literature is available on *Heterostemma* (Costantin 1912; Ho 1993; Li et al. 1995; Tran 2005; Rodda 2016), as well as a recent revision (Tran 2017 [in Vietnamese]), where seven species have been recorded for the country.

While conducting fieldwork in Cuc Phuong National Park, Nho Quan district, Ninh Binh Province in Vietnam, an unidentified species of *Heterostemma* was collected. From the relevant literature (Costantin 1912; Swarupanandan et al. 1989; Ho 1993; Li et al. 1995; Tran 2005, 2017; Tran and Kim 2010; Thaithong et al. 2018; Agnihotri et al. 2019; Kidyoo 2019; Thammarong et al. 2019), as well as an examination of specimens in the herbaria BK, BKF, BM, HN, HNU, HNPM, IBK, IBSC, K, KUN, KYO, P, SING, TI, TO, TUT and VNM (acronyms according to Thiers 2020), we have confirmed that it is a new species. Here, we describe and illustrate this new species as *H. cucphuongense* T.B. Tran and Rodda. We also provide a key to the species of *Heterostemma* that are now known to occur in Vietnam.

## Taxonomy

### Heterostemma cucphuongense

Taxon classificationPlantaeGentianalesApocynaceae

T.B.Tran & Rodda sp. nov.

0A96EDCC-9893-5408-8F5C-2BFBEC6548E5

urn:lsid:ipni.org:names:77209709-1

Fig. 1

#### Diagnostic characters.

This new species is similar to *H. succosum* Kerr, as both species have shortly pedunculate inflorescences, on which the flowers open in gradual succession (with generally a single flower open) and have relatively large rotate flowers (generally > 14 mm diam.). They are separated by the presence of a distinct rachis that develops in the inflorescence (which is absent in *H. succosum*); the margins of the corolla lobes are revolute (vs. flat in *H. succosum*), the pedicels are shorter (5–10 mm, vs. 15–30 mm in *H. succosum*) and by the colour of the adaxial surface of the corolla (red with white-yellow spots vs. yellow-orange with reddish-brown spots in *H. succosum*).

**Figure 1.**
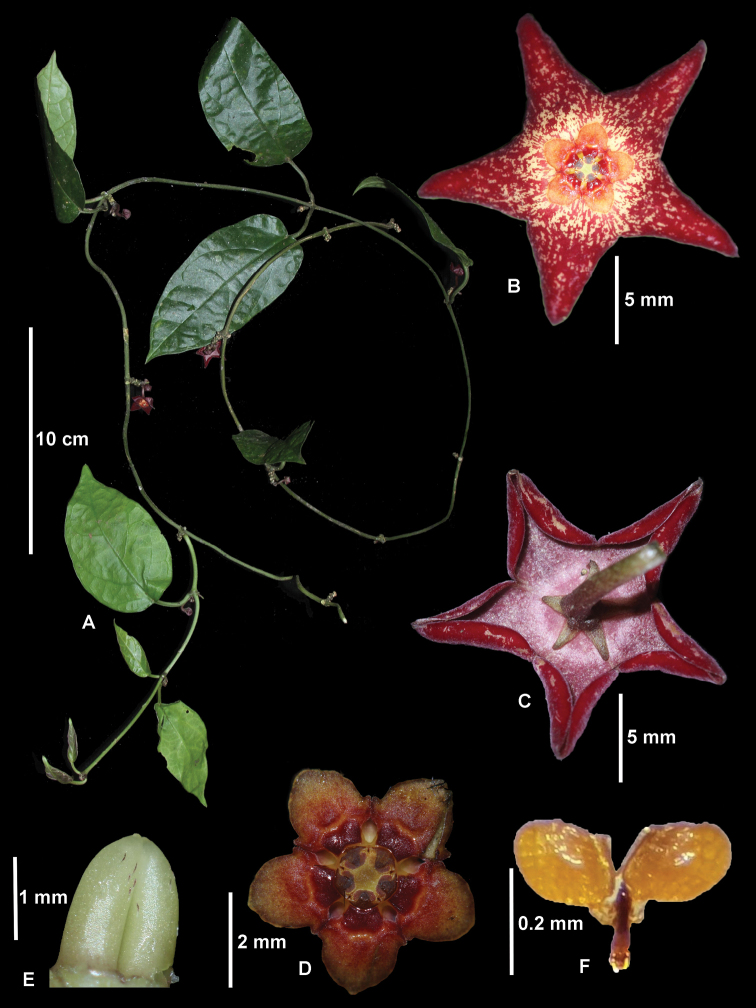
*Heterostemma cucphuongense* T.B.Tran & Rodda **A** flowering branch **B** flower, view from above (adaxial) **C** flower, from underneath **D** corona, from above **E** ovary **F** pollinarium. (Photographs by N.Q. Dat, T.B. Tran, Thuong V.A., photo edit by M. Rodda)

#### Type.

Vietnam. Ninh Binh province, Nho Quan district, Cuc Phuong commune, Cuc Phuong National Park, 5 June 2019, *Le Ngoc Han et al. VB 809* (HN, holotype; HN, isotype).

#### Description.

***Liane***, at least 1 m in length. ***Stem and branches*** pubescent, longitudinally grooved, 1.5–2 mm diam.; internodes 2.5–10 cm long. ***Leaves*: *petiole*** 5–25 × 0.6–2 mm, glabrous or sparely pubescent; ***lamina*** herbaceous, ovate to oblong, 4.5–8.5 × 2–4.5 cm, glabrous above, pubescent below, apex acuminate, base rounded to obtuse, venation pinnate with 3–4 basal secondary veins and 3–6 secondary veins departing from main vein, anastomosing near the edge of the lamina; basal colleters 6–8 at base of lamina; ***Inflorescences*** 1(–8) flowered cymes; ***peduncle*** (0)3–5.5 × 2–2.5 mm, older peduncles developing a rachis to 7 mm long. ***Pedicel*** 5–10 × 1–1.35 mm, brown-red, glabrous or sparsely pubescent. ***Flower*** 1.4–1.9 cm diam. ***Flower buds*** 5-angled, just before anthesis 0.9–1 cm diam. ***Sepals*** deltate, 1.5–2.2 × 0.8–1.2 mm, brownish-green, sparely pubescent on both surfaces; with colleters at sinus. ***Corolla*** rotate, 1.4–1.9 cm diam., red with fine white-yellow spots (becoming more concentrated towards the centre) and glabrous or sparsely pubescent adaxially; bright purple-red and glabrous abaxially; tube 3.4–4.2 × 6.8–8.4 mm; lobes deltate, 5.5–6.7 × 4.5–5.2 mm, margins recurved. ***Corona*** staminal, 4.9–5.2 mm diam., ca. 0.9 mm high, upper surface orange with red centre, lower surface dull red and shiny, glabrous or sparely pubescent; lobes broadly ovate, 1.58–1.66 × 1.10–1.14 mm, with 2 lateral ledges at the junction between adjoining lobes, inner apex acute, outer apex rounded. ***Pollinarium***: pollinia erect, broad elliptic, yellow, 0.2–0.21 × ± 0.16 mm; corpusculum linear lanceolate, brown, ca. 0.18 × 0.04 mm; caudicles ± 0.05 × 0.04 mm; crests translucent, ± 0.12 × 0.02 mm. ***Ovary*** ca. 1.42 × 1.18 mm, greenish-white, sparsely pubescent. ***Fruits and seeds*** not observed.

#### Etymology.

The species is named after the type locality, Cuc Phuong National Park, in Ninh Binh Province, northern Vietnam.

#### Distribution and ecology.

*Heterostemma cucphuongense* was only collected once, near one of the main trails in the Cuc Phuong National Park. It was found in primary evergreen forest on soils derived from degraded limestone. It was collected in flower in June.

#### Conservation status.

*Heterostemma cucphuongense* is endemic to the Cuc Phuong National Park. Since it is known from a single collection, its conservation status is Data Deficient (DD; IUCN 2012).

#### Notes.

*Heterostemma cucphuongense* is similar to *H. succosum*Kerr (1939), a species found in Thailand and Laos. Both have shortly pedunculate inflorescences, in which the flowers generally open one at a time and are relatively large and rotate (mostly > 14 mm diam.). The two species can be easily separated because, in *H. cucphuongense*, the inflorescences form a rachis to 7 mm long with age while the inflorescences of *H. succosum* do not develop any rachis. Furthermore, the pedicels of *H. cucphuongense* are 5–10 mm long, while *H. succosum* has pedicels 15–30 mm long. Further distinguishing characters (that, however, are less obvious in dried material) are the margins of the corolla lobes that are recurved in *H. cucphuongense* (vs. flat in *H. succosum*). The two species also differ in that the colour of the adaxial surface of the corolla is red with white-yellow spots in *H. cucphuongense* (vs. yellow-orange with reddish-brown spots in *H. succosum*). These and additional diagnostic characters separating the two species are listed in Table 1.

**Table 1.** Morphological differences between *Heterostemma cucphuongense* and *H. succosum*.

Characters	*H. succosum*	*H. cucphuongense*
**Length of petiole (cm)**	2.5–7	0.5–2.5
**Shape of leaf blade**	Elliptic	Ovate to oblong
**Length of peduncle (mm)**	4–6	(0)3–5.5
**Rachis**	Absent	Present, to 7 mm long
**Length of pedicel (mm)**	15–30	5–10
**Colour of adaxial surface of corolla**	Yellow-orange with reddish-brown spots	Red with white-yellow spots
**Length of corolla tube (mm)**	4–8	3.4–4.2
**Margins of corolla lobes**	Not recurved	Recurved
**Corona colour**	Brownish-red	Orange with a darker red centre

##### Key to the species of *Heterostemma* in Vietnam

**Table:** 

1	Mature stems developing a corky bark with age	***H. suberosum***
–	Mature stems not becoming covered with a corky bark	**2**
2	Corolla diam. > 6 times corona diam., corona pubescent	***H. xuansonense***
–	Corolla diam. < 4 times corona diam., corona glabrous	**3**
3	Peduncle < 5.5 mm long, stout, 2–3 mm thick	**4**
–	Peduncle > 6 mm long, slender, 1–1.5 mm thick	**5**
4	Pedicels 5–10 mm long	***H. cucphuongense***
–	Pedicels 20–32 mm long	***H. oblongifolium***
5	Corona lobes shorter than corolla tube	**6**
–	Corona lobes as long as or longer than corolla tube	**7**
6	Corona lobes spreading on surface of corolla, almost flat, outer apex simple	***H. brownii***
–	Corona lobes raised from the corolla surface, outer apex trilobed	***H. acuminatum***
7	Peduncle 7–25 mm long, corolla < 8 mm diam.	***H. piperifolium***
–	Peduncle 25–60 mm long, corolla > 10 mm diam.	***H. grandiflorum***

## Supplementary Material

XML Treatment for Heterostemma cucphuongense
